# Prognostic impact of a compartment-specific angiogenic marker profile in patients with pancreatic cancer

**DOI:** 10.18632/oncotarget.2651

**Published:** 2014-12-30

**Authors:** Christoph Kahlert, Maria Fiala, Gabriel Musso, Niels Halama, Sophia Keim, Massimiliano Mazzone, Felix Lasitschka, Mathieu Pecqueux, Fee Klupp, Thomas Schmidt, Nuh Rahbari, Sebastian Schölch, Christian Pilarsky, Alexis Ulrich, Martin Schneider, Juergen Weitz, Moritz Koch

**Affiliations:** ^1^ Department of General, Visceral and Transplantation Surgery, University of Heidelberg, Heidelberg 69120, Germany; ^2^ Department of Medicine, Harvard Medical School, Boston, MA 02115, USA; ^3^ Cardiovascular Division, Brigham and Women's Hospital, Boston, MA 02115, USA; ^4^ Medical Oncology, National Center for Tumor Diseases and Hamamatsu Tissue Imaging and Analysis (TIGA) Center, Institute for Medical Biometry and Informatics, University of Heidelberg, Germany; ^5^ Laboratory of Molecular Oncology and Angiogenesis, Vesalius Research Center, VIB, Leuven 3000, Belgium; ^6^ Laboratory of Molecular Oncology and Angiogenesis, Vesalius Research Center, Department of Oncology, KU, Leuven 3000, Belgium; ^7^ Institute of Pathology, University of Heidelberg, Heidelberg 69120, Germany; ^8^ Department of General, Visceral and Thoracic Surgery, University of Dresden, Dresden 01307, Germany

**Keywords:** Pancreatic cancer, stroma, angiogenic cytokines, expression profile

## Abstract

Pancreatic cancer consists of a heterogenous bulk of tumor cells and stroma cells which contribute to tumor progression by releasing angiogenic factors. Those factors can be detected as circulating serum factors. We performed a compartment-specific analysis of tumor-derived and stroma-derived angiogenic factors to identify biomarkers and molecular targets for the treatment of pancreatic cancer. Kryo-frozen tissue from primary ductal adenocarcinomas (*n* = 51) was laser-microdissected to isolate tumor and stroma tissue. Expression of 17 angiogenic factors (angiopoietin-2, follistatin, GCSF, HGF, interleukin-8, leptin, PDGF-BB, PECAM-1, VEGF, matrix metalloproteinase -1, -2, -3, -7, -9, -10, -12, and -13) was analyzed using a multiplex elisa assay for tissue-derived proteins and corresponding serum.

Our study reveals a compartment-specific expression profile for several angiogenic factors and matrix metalloproteinases. ROC analysis of corresponding serum samples reveals MMP-7 and MMP-12 as strong classifiers for the diagnosis of patients with pancreatic cancer vs. healthy control donors. High expression of tumor-derived PDGF-BB and MMP-1 correlates with prolonged survival in univariate and multivariate analysis. In conclusion, a distinct expression patterns for angiogenic cytokines and MMPs in pancreatic cancer and surrounding stroma may implicate them as novel targets for cancer treatment. Tumor-derived PDGF-BB and MMP-1 are significant and independent prognostic markers for poor survival.

## INTRODUCTION

Pancreatic cancer is the fourth leading cause of cancer-related death in the Western hemisphere [1, 2]. Recent improvements in the treatment of pancreatic cancer by multidisciplinary therapeutic approaches and the introduction of more effective chemotherapy regimens such as the combination of gemcitabine with albumin-bound paclitaxel or the combination of oxaliplatin, irinotecan, fluoruracil, and leucovorin (FOLFIRINOX) have increased significantly the prognosis of a selected group of patients [3–5]. However, the cumulative overall 5-year survival of all patients with pancreatic cancer remains less than 5%, with a median survival of 4 to 6 months [6]. Curative resection through surgery is only possible for approximately 15–20% of cases, and even in these patients, the median survival reaches only 28 months [6].

In an effort to improve the clinical outcome of pancreatic cancer, there is an increasing focus on tailoring therapeutics to individual tumor biology [7]. This involves the use of prognostic biomarkers, including lymph node status, tumor differentiation grade, and tumor size [8–10]. While promising, additional biomarkers are actively sought in an attempt to further stratify patients into different risk groups that might benefit from a more individualized therapy.

In addition to tissue obtained from the primary tumor cells, the adjacent tumor stroma provides an important source of potentially prognostic or predictive biomarkers in tumor disease [11–13]. In these compartments, biomarkers can derive either directly from tumor cells or are indirecte surrogates for the systemic inflammatory response of the host [14, 15]. For example, fibrotic stroma is a major component of the pancreatic tumor bulk [16], consisting of an extracellular matrix and various cell types such as pancreatic stellate cells, endothelial cells and inflammatory cells [16]. The intercellular cross-talk between cancer cells and the tumor microenvironment via angiogenic cytokines or growth factors is essential for tumor progression and dissemination [17]. Moreover, tumor-stroma-associated cells secrete matrix metalloproteinases which can degrade the extracellular matrix and contribute to tumor angiogenesis [18, 19]. Therefore, in addition to tumor epithelial-derived biomarkers, tumor stroma-secreted proteins may supply detailed information about the tumor biology and present novel molecular targets for the treatment of pancreatic cancer [20].

To address this, we examined the suitability of tumor-, stroma- and serum-derived proteins as biomarkers in pancreatic cancer. To do so, we separated cancer cells and surrounding tumor stroma, subsequently comparing the expression of 9 angiogenic cytokines and 8 matrix-metalloproteinases (MMPs) by using the new technology of a multiplex-based angiogenic cytokine and MMP assays. Our study shows a compartment-specific expression profile of angiogenic factors in pancreatic cancer. In corresponding serum samples, we identified serum-derived MMP-7 and MMP-12 as strong classifiers to diagnose patients and tumor-derived PDGF-BB and MMP-1 as potential prognostic biomarkers in pancreatic cancer.

## RESULTS

### Patient characteristics and clinical specimens

51 patients with pancreatic cancer were included in this study and represent a homogenous cohort (Supplementary Table S1). The median age was 67 years, 29 patients were male, 22 female. The UICC stage at time of tumor resection was II in 8 cases, III in 39 cases and IV in 4 cases. Those patients with stage IV pancreatic cancer had either distant lymph node metastases (*n* = 2) or single, small liver metastases (*n* = 2) and had undergone a macroscopically complete tumor resection. Therefore, stage IV patients in our cohort received a similar postoperative treatment and follow-up with a curative attempt as patients with stage II or stage III pancreatic cancer. 28 specimens were diagnosed with a tumor grade of 2, 23 specimens were diagnosed with a tumor grade of 3. Accoring to the definition of the Leeds Pathology Protocol (LEEPP) [21], the tumor was completely resected (resection status R0) in 7 cases, tumor infiltration was proven at the resection margin during pathological analysis of the removed specimen (resection status R1) in 44 cases, none of the patients presented a macroscopic residual disease (resection status R2). One patient did not receive adjuvant treatment as he was not eligible for the inclusion criteria of an adjuvant chemotherapy due to his general state of health (postoperative Karnofsky index < 60%); 50 patients received postoperative chemotherapy.

### Expression profile of MMPs and angiogenic cytokines in serum, cancer and stromal cells

**Figure 1:**
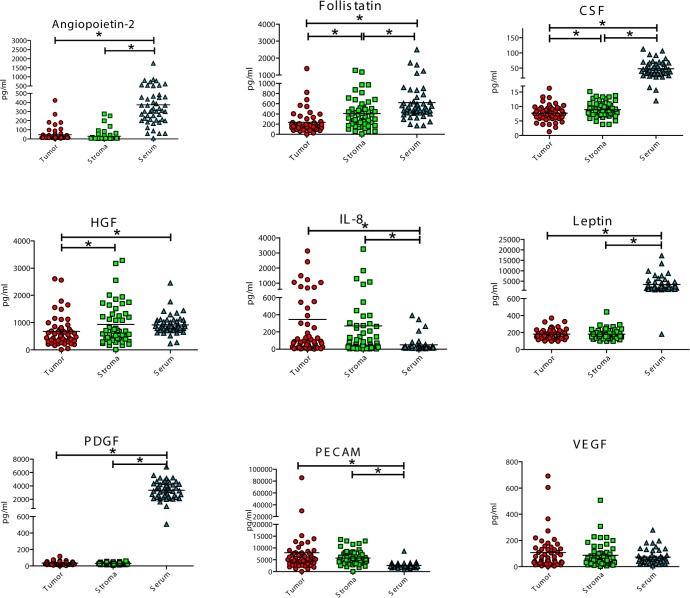

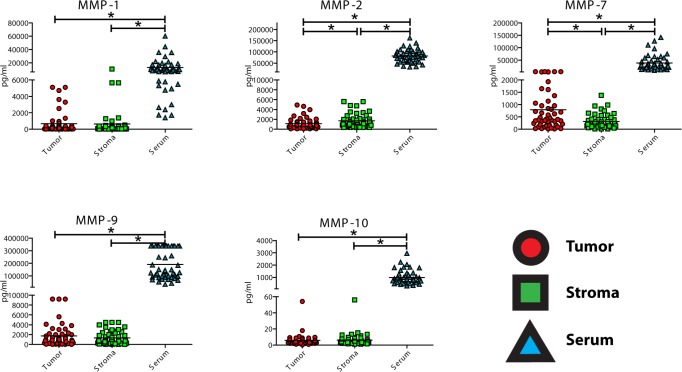
Expression analyis of angiopoietin-2, follistatin, granulocyte colony-stimulating-factor, (G-CSF), hepatocyte growth factor (HGF), interleukin-8 (IL-8, CXCL8), leptin, platelet-dereived growth factor beta (PDGF-BB), platelet endothelial cell adhesion molecule-1 (PECAM-1, sCD31), vascular endothelial growth factor (VEGF). Matrix metalloproteinase (MMP)-1, -2, -7, -9, and -10 in tumor epithelial cells, tumor-associated stroma, and corresponding serum samples. The concentratons are given as pg/ml for each parameter. (*) marks a signficant difference (*p* < 0.05) between the expression of angiogenic cytokines or MMPs among the three different compartments.

Tumor and corresponding stroma and serum samples were examined from each patient in a standardized manner (see Methods). First, we analyzed the expression of 9 angiogenic cytokines and 8 MMPs in tumor cells, tumor stroma and serum of the same patient (Figure 1, Supplementary Table S2). In 1 of the 51 tissue samples and 3 of 51 serum samples, the multiplex measurement failed and data were excluded from further analysis. Additionally, MMP-3, MMP-12, and MMP-13 could only be detected in fewer than 25% of the tissue samples, and thus these MMPs were excluded from further correlation analysis. Analysis of angiogenic markers from all three compartments (tumor cells, stroma cells, and serum) revealed high serum expression for five angiogenic cytokines and five MMPs as compared to tumor and stroma tissue (*p* < 0.001; Figure 1): angiopoietin-2, follistatin (tumor only), CSF, leptin, PDGF-BB, MMP-1,-2,-7,-9 and -10. Serum-derived PECAM-1 displayed a significantly lower expression in serum, as compared to stroma tissue (*p* < 0.001). Alternately, MMP-7 had increased expression in tumor tissue as compared to stroma (*p* < 0.001). Follistatin was significantly upregulated in tumor stroma, as compared to tumor cells (*p* < 0.001) while G-CSF, HGF and MMP-2 showed a trend for an increased expression in the stromal compartment. Pearson correlation between serum-derived factors and tissue-derived factors revealed no significant linear relationship for any angiogenic cytokine nor any MMP (Supplementary Table S3, Figure 1).

### Evaluation of serum MMPs as pancreatic cancer diagnostic biomarkers

**Figure 2:**
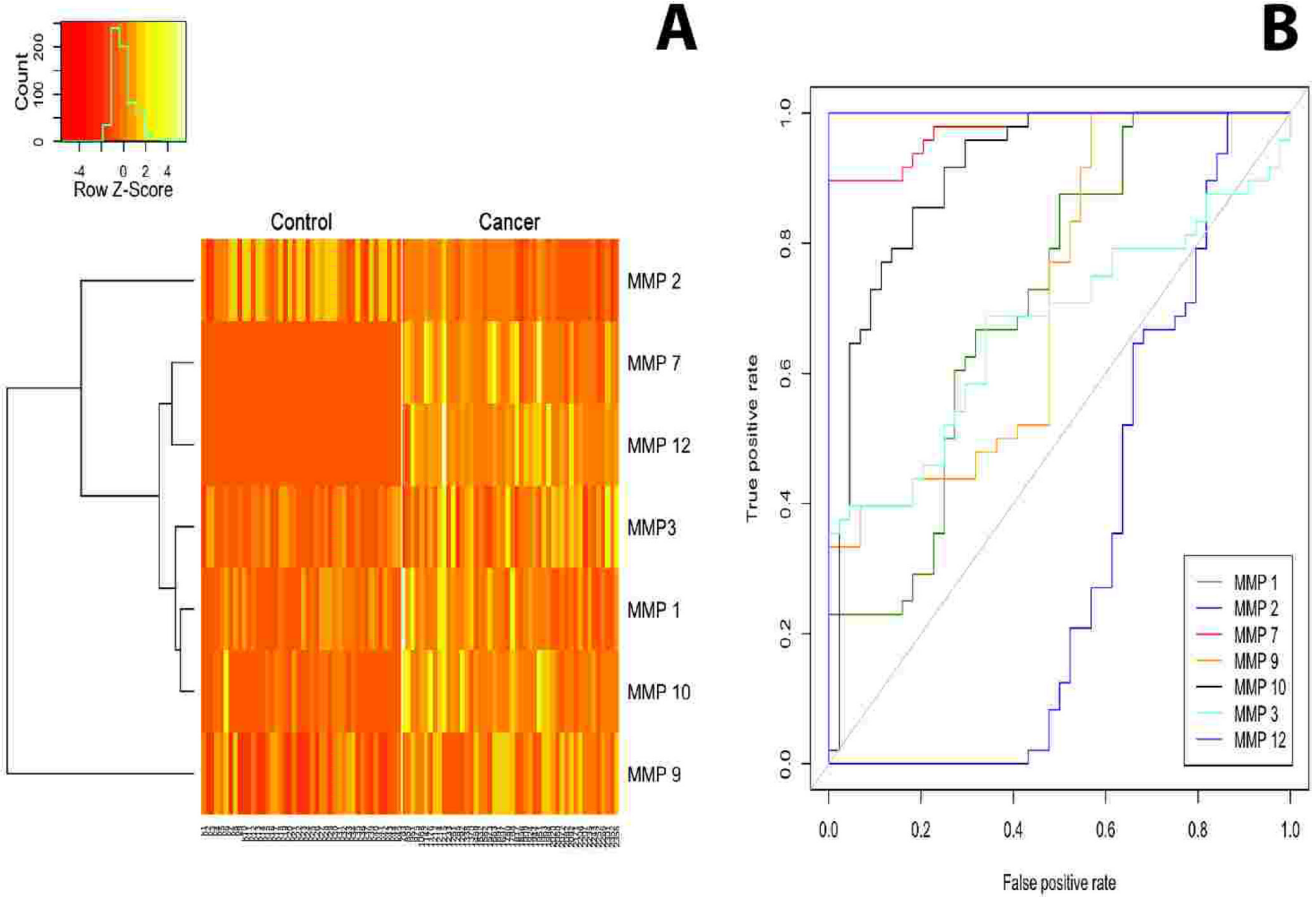
(A) The heatmap displays the expression pattern of serum MMPs in samples from healthy donors (control) and patients with pancreatic cancer MMP-2 was significantly downregulated in PDAC serum samples compared to control (*p* > 0.0001), whereas MMP-1,-3,-7,-9,-10 and -12 were significantly increased in cancer patients (*p* > 0.0001). **(B)** Area under the receiver operating characteristic (ROC) curves for all MMPs with samples from patients with pancreatic cancer and the control group.

The role of angiogenic cytokines as potential serum biomarkers to distingish between patients with pancreatic cancer and healthy donors has already been adressed [12, 22]. Therefore, we focused the next phase of our analysis on the circulating MMPs as potential diagnostic markers to discriminate between patients with pancreatic cancer and healthy individuals. As control cohort, serum samples of 44 healthy donors were included. MMP-1,-3,-7,-9,-10 and -12 were significantly upregulated in patients with pancreatic cancer (*p* < 0.0001, respectively), whereas MMP-2 was significantly decreased in the cancer patient group (*p* < 0.0001; Figure 2A). While all MMPs served as good predictors of disease, as assessed using area under the receiver operating characteristic curve (AUC) (see Table 1), MMP-7 and MMP-12 are perfect classifiers (MMP-7: AUC = 0.97, sensitivity = 90%, specificity = 100% MMP-12: AUC = 1, sensitivity = 100%, specificity = 100%), suggesting them as meaningful diagnostic biomarkers in pancreatic cancer. Subgroup ROC analysis of stage II – IV pancreatic cancer revealed similar results for MMP-7 and MMP-12 (Supplementary Table S4).

**Table 1:** Area under the receiver operating characteristic curves (AUC) of serum MMPs for the diagnosis of patients with pancreatic cancer vs. healthy control donors The cutoff was determined using the Youden-Index.

Serum marker	AUC	Cutoff	Sensitivity	Specificity
MMP-1	0.71	7754.2	0.67	0.68
MMP-2	0.34	75210.8	0.65	0.34
MMP-3	0.67	14787.72	0.69	0.66
MMP-7	0.97	12324.5	0.9	1
MMP-9	0.71	90602.1	0.77	0.52
MMP-10	0.91	559.5	0.85	0.82
MMP-12	1	2215.58	1	1

### Compartment-specific angiogenic marker profile of MMPs and angiogenic cytokines in correlation to clinicopathological parameters and clinical outcome

To evaluate the prognostic relevance of tumor-derived, stroma-derived, and serum-derived angiogenic markers, the expression of each factor was correlated with clinical outcomes and histopathological parameters. The patient cohort was divided into groups based on expression, such that those having expression of angiogenic cytokines or MMPs below the median were considered low, and all else considered high. Chi square tests revealed no statistically significant correlation between any tumor-derived, stroma-derived or serum-derived angiogenic factor (Supplementary Table 5 – 11). Unsupervised hierachical clustering based on expression of angiogenic cytokines and MMPs did not reveal any distinct subsets of patients for tumor-, serum-, or stroma-derived factors (Supplementary figure 1). Similarly, no distinct clusters were apparent when supervising cluster analysis based on patient tumor grade or UICC stage of the primary cancer (data not shown).

**Figure 3:**
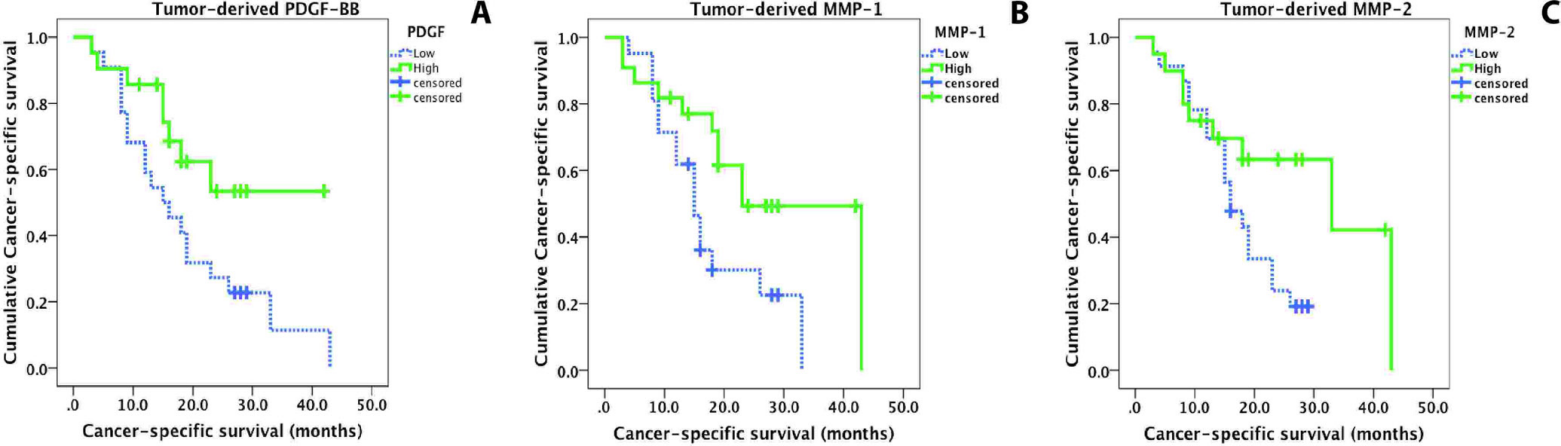
Kaplan-Meier curves display cancer-specific survival in patients with pancreatic cancer. Solid line: Low expression; dashed line: high expression **(A)** Overexpression of tumor-derived PDGF-BB is significantly associated with prolonged survival (log-rank test, *p* = 0.038). **(B)** Overexpression of tumor-derived MMP-1 is significantly associated with prolonged survival (log-rank test, *p* = 0.029). **(C)** Overexpression of tumor-derived MMP-2 is an indicator for a better prognosis and shows a trend for an improved clinical outcome (*p* = 0.058).

Alternately, univariate analysis (see Methods) revealed that the expression of three angiogenic factors was predictive of survival in our patient cohort (*p* < 0.05; Figure 3 & Table 2). Specifically, high expression of tumor-derived PDGF-BB and MMP-1 were associated with increased cancer survival (*p* < 0.05), while high expression of MMP-2 showed a trend towards improved outcome (*p* = 0.058) (see Table 2). Notably, survival analysis did not show any significant associations between expression of any stroma- or serum-derived angiogenic cytokine or MMP and clinical outcome. Using multivariate testing (Table 3), which includes all relevant clinicopathological parameters as well as all angiogenic cytokines and MMPs, high expression of tumor-derived PDGF-BB and high expression of tumor-derived MMP-1 were independent prognostic markers for cancer-specific survival (PDGF-BB: Odds ratio: 0.265, CI: 0.101-0.693, *p* = 0.007; MMP-1: Odds ratio: 0.347, CI: 0.121-0.996, *p* = 0.049).

**Table 2:** Univariate analysis (log-rank test) of 9 angiogenic cytokines and 5 MMPs for median cancer-specific survival Samples were grouped as low (≤ median expression of angiogenic cytokines or MMPs) or high (> median expression of angiogenic cytokines or MMPs) level of expression and were analyzed separately from pancreatic tumor cells (tumor), surrounding tumor stroma (stroma) and corresponding serum.

Cytokines/MMPs	Compartment	Cancer-specific Survival (Months)		*p*-Value
		Low	High	
**Angiopoietin-2**	Tumor	18.0	19.0	0.92
	Stroma	18.0	19.0	0.41
	Serum	19.0	15.0	0.36
**Follistatin**	Tumor	23.0	18.0	0.59
	Stroma	23.0	16.0	0.789
	Serum	16.0	23.0	0.4
**G-CSF**	Tumor	18.0	23.0	0.37
	Stroma	19.0	19.0	0.99
	Serum	19.0	18.0	0.417
**HGF**	Tumor	18.0	23.0	0.46
	Stroma	33.0	19.0	0.35
	Serum	18.0	23.0	0.537
**IL-8**	Tumor	18.0	23.0	0.59
	Stroma	18.0	19.0	0.41
	Serum	16.0	23.0	0.495
**Leptin**	Tumor	19.0	18.0	0.37
	Stroma	23.0	16.0	0.26
	Serum	19.0	16.0	0.301
**PDGF-BB**	Tumor	15.0	Median not reached	***0.038***
	Stroma	18.0	19.0	0.26
	Serum	18.0	26.0	0.356
**PECAM-1**	Tumor	16.0	23.0	0.57
	Stroma	26.0	15.0	0.19
	Serum	18.0	26.0	0.257
**VEGF**	Tumor	18.0	23.0	0.49
	Stroma	16.0	43.0	0.26
	Serum	19.0	43.0	0.573
**MMP-1**	Tumor	15.0	23.0	**0.029**
	Stroma	16.0	19.0	0.53
	Serum	19.0	15.0	0.122
**MMP-2**	Tumor	16.0	33.0	*0.058*
	Stroma	19.0	18.0	0.83
	Serum	19.0	15.0	0.136
**MMP-7**	Tumor	16.0	23.0	0.32
	Stroma	16.0	19.0	0.38
	Serum	19.0	15.0	0.320
**MMP-9**	Tumor	16.0	43.0	0.074
	Stroma	19.0	19.0	0.71
	Serum	23.0	13.0	0.182
**MMP-10**	Tumor	23.0	16.0	0.133
	Stroma	19.0	18.0	0.49
	Serum	19.0	18.0	0.459

**Table 3:** Multivariate analysis (Cox proportional hazards regression model) of prognostic parameters for cancer-specific overall survival in pancreatic cancer (CI: confidence interval).

Characteristics	Category	Hazard ratio	95% CI of Relative Risk	*P*-Value
**Gender**	(Male/Female)	1.728	0.576 – 5.181	0.329
**MedianAge**	≤ 70 years vs. > 70 years	0.369	0.144 – 0.949	***0.039***
**T-stage**	pT3 vs. pT4	0.165	0.017 – 1.579	0.118
**N-stage**	pN0 vs. pN1	2.344	0.652 – 8.426	0.192
**M-stage**	M0 vs. M1	1.033	0.197 – 5.415	0.97
**Tumorgrade**	G2 vs. G3	2.787	0.96 – 8.094	0.06
**Resection status**	R0 vs. R1	1.322	0.284 – 6.152	0.722
**Tumor PDGF**	Low vs. high expression	0.265	0.101 – 0.693	***0.007***
**Tumor MMP-1**	Low vs. high expression	0.347	0.121 – 0.996	***0.049***
**Tumor MMP-2**	Low vs. high expression	0.616	0.208 – 1.828	0.383

## DISCUSSION

Pancreatic cancer is composed of a heterogenous bulk of tumor cells and adjacent stroma cells, with both of these compartments regulating tumor progression and dissemination through the release of angiogenesis-associated cytokines and matrix-metalloproteinases [17].

In this context, the functional role of the tumor stroma remains ambiguous. Some components of the tumor stroma such as activated cancer-associated pancreatic stellate cells are implicated in neoangiogenesis by activating the c-MET pathway through the release of HGF [23]. In contrast, Rhim et al. have shown that hedgehog-driven stroma suppresses tumor growth in part by restraining tumor angiogenesis [24].

Our study presents a detailed expression profile of angiogenic biomarkers in pancreatic cancer cells, as well as surrounding tumor stroma and corresponding serum samples. These findings reflect the dichotomous stimulating and inhibiting roles of tumor stroma in pancreatic cancer: consistent with previous reports [25, 26], our data show a tendancy that tumor-adjacent stroma is the major source of several pro-angiogenic cytokines and MMPs (*e.g.* HGF, MMP-2, and G-CSF). Likewise, the stroma component displayed a significantly enhanced release of follistatin, which can suppress the formation of multiple organ metastasis predominantly by inhibiting angiogenesis [27]. These findings support the hypothesis that pancreatic tumor stroma is not uniform but its cellular heterogeneity can exert tumor-promoting as well as host-protective functions by impairing neovascularization. In this context, it can be conjectured that the hypoxic and hypovascular microenvironment in pancreatic cancer [28, 29] is a surrogate marker for the host defense mechanism of the host tissue against cancer cells. However, from a therapeutical point of view, a strong desmoplastic and hypovascular tumor-associated stroma is a double-edged sword: on the one hand it may constitute a protective response from the host against tumor cells and act as a physical barrier against tumor cell invasion [30]. On the other hand, the hypovascular stroma impairs the delivery of cytotoxic chemotherapies to the peritumoral milieu [30, 31]. This might be one reason that the combination of standard chemotherapies such as gemcitabine with antiangiogenic therapies such as bevacizumab have failed to improve the outcome in patients with pancreatic cancer [32].

Additionally, since circulating MMPs and angiogenic cytokines have been proposed as relevant non-invasive surrogate markers in tumor disease [33–35], we compared compartment-specific expression between serum, cancer cells and stromal cells of the same patient. An unsupervised hierachical clustering based on the expression of angiogenic cytokines and MMPs did not reveal any distinct subsets of patients for tumor-, serum-, or stroma-derived factors. Similarly, no distinct clusters were apparent when supervising cluster analysis based on patient tumor grade or UICC stage of the primary cancer. Based on these findings, one may assume that a panel of 17 angiogenic factors is not specific enough to identify molecular subgroups of pancreatic cancer which have a distinct expression profile. To determine different biological subtypes of pancreatic cancer, other tools such as RNA expression analysis or RNA sequencing might be preferred as they have a more informative value to adress this question [36–38]. Furthermore, we found the majority of angiogenic cytokines and MMPs to be more highly expressed in serum, albeit without any significant linear relationship between pancreatic cancer tissue and serum for any angiogenic cytokine or MMP. These results are consistent with previous reports investigating the correlation between tissue tumor biomarkers and corresponding serum samples [39, 40]. Our findings could be explained by the presence of immunocompetent cells in the blood, which are a known source of circulating angiogenic cytokines and MMPs [41]. In light of this, serum-derived biomarkers do not only reflect the expression profile of the solid tumor, but also represent the inflammatory host response. This qualifies circulating angiogenic factors as being useful surrogate markers for tumor diagnosis and prognosis. However, due to the heterogenous source of circulating factors, they offer limited insight into tumor biology itself. This may also explain why the unsupervised cluster analysis of angiogenic serum markers did not reveal any specific subgroups which were associated with a more aggressive tumor type or a more advanced tumor stage.

Consistent with their presumptive role as biomarkers, we have identifed MMP-1,-3,-7,-9,-10 and 12 as being upregulated in the serum of patients with pancreatic cancer, as compared to a cohort of healthy donors. Among those, MMP-7 and MMP-12 displayed high sensitivity and specificity in discriminating between serum samples of pancreatic cancer patients and healthy donors. Even in a small subgroup of patients with early stage pancreatic cancer (UICC II), MMP-7 and MMP-12 were good classifiers to distinguish between patients with pancreatic cancer and healthy donors. These findings advocate MMPs as having a high potential as diagnostic serum markers for pancreatic cancer. Moreover, they may offer a more cost-effective and simply applicable alternative with a similar diagnostic value in comparison to other fluid biomarkers such as microRNAs [42]. However, further studies are required to unravel the question of whether elevated serum MMPs are specific for pancreatic cancer, or are increased in other tumor types or chronic diseases as well.

To assess wether these proteins could predict more specific disease properties, we assessed the compartment-specific expression profiles of angiogenic cytokines and MMPs in correlation with histopathological data and disease prognosis. Our findings indicated that while tumor-derived angiogenic cytokines and MMPs are potential prognostic biomarkers, stroma- and serum-derived factors did not show any significant association between expression level and survival. Low expression of tumor-derived PDGF-BB correlated significantly with a decreased cancer-specific survial. PDGF-BB activates pericytes by the tyrosine kinase receptor PDGFRβ [43, 44], which likely serve as important gatekeepers against cancer progression and metastasis by stabilizing the tumor vasculature [45]. Previous data has shown that increasing the pericyte content of the pancreatic tumor microenvironment inhibits the growth of angiogenesis-dependent tumors [43] and that poor pericyte coverage increases hypoxic strain in breast cancer, which activates epithelial-to-mesenchymal transition and enhances metastasis [45]. Thus, we hypothesize that overexpression of tumor-derived PDGF-BB results in an increased activation of pericytes in the pancreactic cancer-associated vasculature, inhibiting the spread of tumor cells and improving prognosis.

Alternately, tumor-derived MMP-1 and -2 were associated with a prolonged survival, with MMP-1 being a prognostic factor in both the univarate and multivariate survival analysis. Though several MMPs have been claimed to contribute to tumor dissemination by supporting tumor cell vascular penetration, more recent experimental data indicate that some members of the MMP family can also exert tumor-suppressive functions [46–48]. For example, Wong *et al.* have observed that MMP1 expression was downregulated with advancing disease stage for both colon and rectal cancers [46]. Moreover, this study reported that patients with stage III colon cancer experienced shorter time to distant metastasis and decreased overall survival when lacking MMP-1 [46]. Furthermore, by performing a large meta-analysis of colorectal cancer high-throughput gene-expression studies, Wong *et al.* identified MMP2 as being lesser expressed in colorectal metastases, as compared to the primary tumor. The authors provided evidence that inhibition of MMP2 promotes cell invasion *in vitro*, an effect most dramatic in chemoresistant cells [46]. Thus, while the potential anti-tumorigenic role of MMP-1 and -2 requires further investigation, our findings might explain why many clinical trials in the last decade have failed when broad-spectrum MMP-inhibitors were applied to patients in an attempt to find an anticancer agent [49].

In conclusion, our study exhibits a new approach to detect potential biomarkers and therapeutic molecluar targets in pancreatic cancer. We show that the tumor-associated stroma is a major source of various angiogenic cytokines and MMPs. Moreover, we demonstrate that a panel of circulating serum MMPs is a useful tool in primary tumor to discriminate between healthy donors and patients with pancreatic cancer. These findings require further validation in subsequent clinical trials but might be helpful to detect pancreatic cancer at an early stage, hence improving the early prognosis of the disease. Finally, we have identified tumor-derived PDGF-BB and MMP-1 as novel prognostic markers. Further clinical trials may validate these factors as useful biomarkers to classify patients into different risk groups that might benefit from a more personalized therapy.

## MATERIALS AND METHODS

### Patient characteristics and data collection

The use of patient tissue samples and clinicopathological information in this study has been approved by the Medical Ethical Committee of the University of Heidelberg. Written informed consent was obtained from each patient preoperatively and from each healthy donor prior to serum collection. Kryo-frozen tissue samples were obtained from patients with primary pancreatic adenocarcinomas who underwent tumor resection between 2007 and 2011 at the Department of General, Visceral, and Transplantation Surgery, University of Heidelberg. Clinical information included age, gender, UICC stage, grading, resection status and cancer-related survival (time from diagnosis to cancer-related death or last follw-up).

### Clinical specimens

Kryo-frozen tumor samples were retrieved as previously described [50]. Briefly, samples were snap-frozen in liquid nitrogen immediately after resection and stored at –80°C. A 10μm reference section of each sample was cut and stained with hematoxylin and eosin (standard methods) to indicate the proportion of tumor tissue and adjacent tumor stroma. Serum samples of patients with pancreatic cancer were obtained by taking blood samples immediately before surgical incision while serum samples from healthy donors were obtained under standard sterile conditions. The blood samples were then centrifuged at 2,500g for 10 minutes to extract the serum, which was then stored at –80°C until further analysis.

### Microdissection

#### Tissue preparation and laser microdissection

Tissue preparation for laser microdissection was performed as previously described [51]. A 10 μm reference section of each sample was cut and stained with hematoxylin and eosin by standard methods to evaluate the proportion of tumor tissue and adjacent tumor stroma. Samples with a tumor stroma proportion > 30 % were included into this study. Briefly, 20 μm sections of kryo-frozen pancreatic cancer tissue were mounted on Zeiss membrane slides (Carl Zeiss microimaging, Jena, Germany), stained with cresyl violet using a LCM Staining Kit (Ambion^®^/Applied Biosystems, Darmstadt, Germany) and stored at –80°C until further processing. A calculated area of 40 mm^2^ of tumor epithelial tissue and 40 mm^2^ of surrounding tumor stroma were obtained separately by laser microdissection (PALM Microbeam, Carl Zeiss microimaging, Jena, Germany) and stored in an adhesive cap (Carl Zeiss, Jena, Germany) immediately at –80°C until further processing.

#### Protein lysates

Tissue samples were lysed in Bio-Plex Lysis Buffer, and protein concentrations determined using a BCA protein assay kit (Thermo Scientific, 58239 Schwerte, Germany). Lysates were adjusted to a total protein concentration of 150 μg/ml and 7.5 μg protein (50 μL). Serum samples from both tumor patients and controls were centrifuged and either diluted 1:20 with assay buffer (for panel 1 of the MMP-assay) or used undiluted (for the other assays) according to the manufacturer's instructions. 40 μl of the diluted and undiluted serum samples were transferred into a 96-well plate and immediately analyzed. Microdissected tissue and serum samples were quantified using the Bio-Plex Human Angiogenesis Assay (Bio-Rad Laboratories, Inc., Hercules, CA 94547 USA) and the Millipore MILLIPLEX MAP Human MMP Panel 1 and 2 (Millipore 290 Concord Road, Billerica, MA) according to the manufacturer's instructions and as described recently [13]. These panels included the following proteins: angiopoietin-2, follistatin, granulocyte colony-stimulating-factor (G-CSF), hepatocyte growth factor (HGF), interleukin-8 (IL-8, CXCL8), leptin, platelet-derived growth factor beta (PDGF-BB), platelet endothelial cell adhesion molecule-1 (PECAM-1, sCD31), vascular endothelial growth factor (VEGF), and matrix metalloproteinases (MMPs)-1, -2, -3, -7, -9, -10, -12, and -13. Standard curves and concentrations were calculated with Bio-Plex Manager 4.1.1 using a 5-parameter logistic regression formula.

### Statistical analysis

The SPSS software package version 21.0 (Chicago, IL, USA) and the R statistical framework, version 2.15 (http://www.r-project.org) were used for all calculations. Pairwisse Student t tests were performed to compare the expression of angiogenic factors in pancreatic cancer cells, tumor stroma and corresponding serum and Bonferroni correction was applied to allow for multiple testing. Correlations between the expression of tissue-derived and serum-derived factors were determined using Pearson's correlation. Positive correlations are reported at a Pearson's coefficient (*r*) > 0.5, while inverse correlation are at *r* < −0.5. Chi squared (χ^2^) tests were applied to examine correlation between the expression of angiogenic factors and clinical and pathological parameters and Bonferroni correction was applied to allow for multiple testing. Heatmaps were drawn using the enhanced heatmap package (http://hosho.ees.hokudai.ac.jp/~kubo/Rdoc/library/gplots/html/heatmap.2.html), and area under the receiver operating characteristic curve (AUC)/sensitivity analysis was done using the ROCR package [52], both for R. Univariate survival analysis was performed using the Kaplan-Meier method, with survival differences calculated with the log-rank test. Multivariable analysis (Cox proportional hazards regression model) of cancer-specific survival was performed on all covariates that showed significant association with all relevant clinicopathological parameters in the univariate analysis.

## SUPPLEMENTARY FIGURE AND TABLES



## Abbreviations

G-CSF: granulocyte colony-stimulating-factor
HGF: hepatocyte growth factor
IL-8: interleukin-8
PDGF-BB: platelet-derived growth factor beta
PECAM-1: platelet endothelial cell adhesion molecule-1
VEGF: vascular endothelial growth factor
MMP: matrix metalloproteinase.
