# Supplementary material for: Prognostic impact of a compartment-specific angiogenic marker profile in patients with pancreatic cancer
Source: Oncotarget. 2014 Dec 30;5(24):12978–89. doi: 10.18632/oncotarget.2651 (PMC4350362; doi:10.18632/oncotarget.2651)
Supplement: Supplementary file 1 [file oncotarget-05-12978-s001.pdf]

SUPPLEMENTARY FIGURE AND TABLES

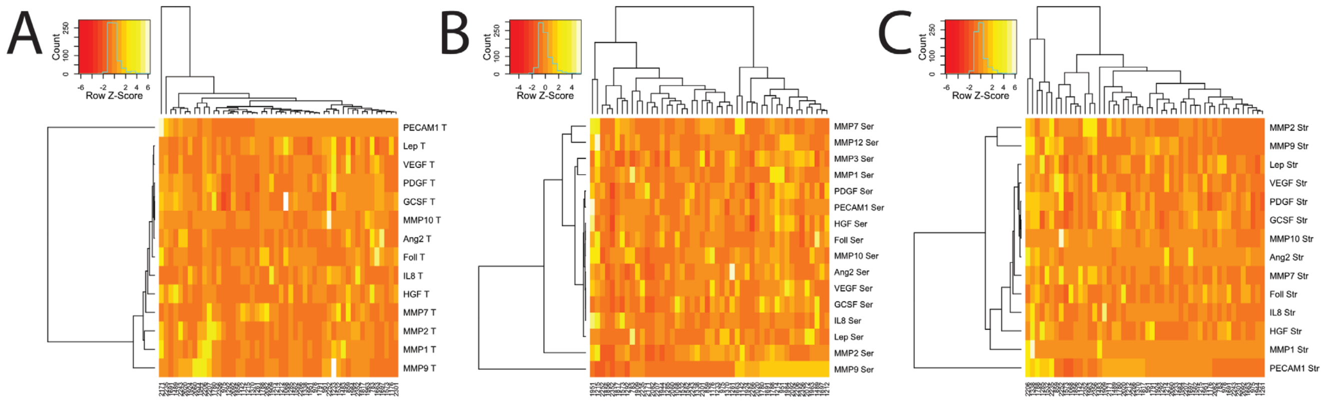

Supplementary Figure S1: The heatmap displays an unsupervised hierachical cluster analysis to identify potential subgroups of patients depending on their expression pattern of angiogenic cytokines and MMPs, grouped into **(A)** tumor-derived factors, **(B)** stroma-derived factors and **(C)** serum-derived factors.

Supplementary Table S1. Summary of clinical parameters of patients included in this study.

Prognostic parameters	Total number of cases
**Age**	
≤ median (67 years)	26
> median (67 years)	25
**Gender**	
Male	29
Female	22
**UICC stage**	
I	0
II	8
III	39
IV	4
**pT stage**	
1	0
2	0
3	50
4	1
**pN stage**	
0	9
1	42
**pM stage**	
0	47
1	4
**Grading**	
1	0
2	28
3	23
4	0
**Resection status**	
R0	7
R1	44
R2	0
**Adjuvant chemotherapy**	
Not received	1
Received	50

Supplementary Table S2. Average expression (mean value; *n* = 49) of 9 angiogenic cytokines and 5 matrix metalloproteinases (MMPs) in pancreatic tumor cells, adjacent tumor stroma and corresponding serum samples of the same patient. Units: pg/ml. *P*-values of all statistical tests were two-sided and $\rho \leq 0.001$ (after Bonferroni correction) were considered to be significant.

Cytokines/ MMPs	Compartment			*p*-Value		
	Tumor Tissue (T)	Stroma Tissue (S)	Serum (Ser)	Tumor vs. Stroma	Tumor vs. Serum	Stroma vs. Serum
**Angiopoietin-2**	45.24	29.65	375.58	0.127	***0,0000000006***	***0,0000000001***
**Follistatin**	234.8	408.15	622.42	**0,0001**	***0,000005***	0.007
**G-CSF**	7.67	8.88	48.01	0.003	***0,0000000001***	***0,0000000001***
**HGF**	680.99	938.87	923.23	0.009	0.02	0.718
**IL-8**	346.29	272.04	48.66	0.190	0.002	0.01
**Leptin**	181.157	179.84	3393.47	0.881	***0,0000000001***	***0,0000000001***
**PDGF-BB**	34.77	30.95	3355.88	0.12	***0,0000000001***	***0,0000000001***
**PECAM-1**	7938.32	5757.63	2599.8	0.20	***0.005***	***0,00000008***
**VEGF**	106.82	87.19	73.26	0.140	0.084	0.234
**MMP-1**	669.66	636.97	12883.15	0.897	***0,000000004***	***0,000000003***
**MMP-2**	1101.93	1681.01	81923.9	0.004	***0,0000000001***	***0,0000000001***
**MMP-7**	790.39	315.44	38137.4	***0.0002***	***0,0000000001***	***0,0000000001***
**MMP-9**	1741.26	1318.30	190912	0.145	***0,0000000001***	***0,0000000001***
**MMP-10**	5.8	6.16	988.25	0.396	0,0000000001	0,0000000001

Supplementary Table S3. The Pearson correlation was employed for determining a correlation between the expression of tissue-derived factors and serum-derived factors. A positive correlation was considered with a coefficient > 0.5, an inverse correlation was considered with a coefficient < - 0.5. *P*-values of all statistical tests were two-sided and $\rho \leq 0.001$ (after Bonferroni correction) were considered to be significant.

	**Pearson correlation coefficient Tumor vs. Stroma**	***p*-value**	**Pearson correlation coefficient Tumor vs. Serum**	***p*-value**	**Pearson correlation coefficient Stroma vs. Serum**	***p*-value**
**Angiopoietin-2**	0.482	<0.0001	0.048	0.747	0.082	0.585
**Follistatin**	0.402	0.004	-0.116	0.436	-0.041	0.785
**CSF**	0.459	0.001	0.203	0.176	0.179	0.229
**HGF**	***0.511***	***<0.0001***	0.021	0.890	0.011	0.940
**IL-8**	***0.778***	***<0.0001***	-0.129	0.387	-0.09	0.544
**Leptin**	0.476	0.001	0.20	0.178	0.162	0.275
**PDGF-BB**	0.481	<0.0001	0.047	0.752	0.035	0.815
**PECAM-1**	0.233	0.103	-0.042	0.779	-0.027	0.857
**VEGF**	***0.735***	***<0.0001***	-0.042	0.778	-0.027	0.857
**MMP-1**	0.388	0.005	-0.084	0.573	0.049	0.745
**MMP-2**	0.418	0.002	-0.359	0.013	-0.293	0.046
**MMP-7**	0.410	0.003	0.044	0.771	-0.032	0.829
**MMP-9**	0.449	0.001	-0.017	0.913	-0.005	0.971
**MMP-10**	***0.935***	***<0.0001***	-0.131	0.380	-0.097	0.515

Supplementary Table S4. UICC tumor stage-dependent area under the receiver operating characteristic curves (AUC) of serum MMP-7 and MMP-12 for the diagnosis of patients with pancreatic cancer vs. healthy control donors. The cutoff was determined using the Youden-Index.

Serum marker		AUC	Cutoff	Sensitivity	Specificity
MMP-7	UICC II	1	12324,53	1	1
MMP-7	UICC III	0,97	12324,53	0,93	1
MMP-7	UICC IV	0,97	12324,53	0,92	1
MMP-12	UICC II	1	2215.58	1	1
MMP-12	UICC III	1	2215.58	1	1
MMP-12	UICC IV	1	2215.58	1	1

Supplementary Table S5. Chi square (χ^2) test to evaluate an association between median age and angiogenic cytokines/MMPs derived from tumor cells, tumor-associated stromal cells or serum. *P*-values of all statistical tests were two-sided and $p \leq 0.001$ (after Bonferroni correction) were considered to be significant.

Cytokines/MMPs	Compartment	Median Age		*p*-Value
		≥ median age	< median age	
**Angiopoietin-2**	Tumor **Low**	17	13	0.25
	Tumor **High**	8	12	
	Stroma **Low**	18	8	0.005
	Stroma **High**	7	17	
	Serum **Low**	10	14	0.39
	Serum **High**	13	11	
**Follistatin**	Tumor **Low**	13	12	0.77
	Tumor **High**	12	13	
	Stroma **Low**	14	11	0.39
	Stroma **High**	11	14	
	Serum **Low**	14	10	0.15
	Serum **High**	9	15	
**G-CSF**	Tumor **Low**	11	14	0.32
	Tumor **High**	14	11	
	Stroma **Low**	12	13	0.77
	Stroma **High**	13	12	
	Serum **Low**	13	11	0.39
	Serum **High**	10	14	
**HGF**	Tumor **Low**	11	14	0.40
	Tumor **High**	14	11	
	Stroma **Low**	10	15	0.16
	Stroma **High**	15	10	
	Serum **Low**	11	13	0.77
	Serum **High**	12	12	
**IL-8**	Tumor **Low**	11	14	0.39
	Tumor **High**	14	11	
	Stroma **Low**	12	13	0.77
	Stroma **High**	13	12	
	Serum **Low**	14	10	0.15
	Serum **High**	9	15	

(*Continued*)

Cytokines/MMPs	Compartment	Median Age		p-Value
		≥ median age	< median age	
**Leptin**	Tumor **Low**	13	12	1.00
	Tumor **High**	12	13	
	Stroma **Low**	10	11	0.26
	Stroma **High**	15	14	
	Serum **Low**	14	11	0.24
	Serum **High**	9	14	
**PDGF-BB**	Tumor **Low**	12	13	0.77
	Tumor **High**	13	12	
	Stroma **Low**	11	14	0.40
	Stroma **High**	14	11	
	Serum **Low**	12	12	0.77
	Serum **High**	11	13	
**PECAM-1**	Tumor **Low**	12	13	0.77
	Tumor **High**	13	12	
	Stroma **Low**	10	15	0.16
	Stroma **High**	15	10	
	Serum **Low**	12	12	0.77
	Serum **High**	11	13	
**VEGF**	Tumor **Low**	15	10	0.16
	Tumor **High**	10	15	
	Stroma **Low**	14	11	0.40
	Stroma **High**	11	14	
	Serum **Low**	12	12	0.77
	Serum **High**	11	13	
**MMP-1**	Tumor **Low**	12	13	0.77
	Tumor **High**	13	12	
	Stroma **Low**	10	15	0.16
	Stroma **High**	15	10	
	Serum **Low**	14	10	0.15
	Serum **High**	9	15	
**MMP-2**	Tumor **Low**	11	15	0.26
	Tumor **High**	14	10	
	Stroma **Low**	11	14	0.40
	Stroma **High**	14	11	
	Serum **Low**	15	9	0.043
	Serum **High**	8	16	

(*Continued*)

Cytokines/MMPs	Compartment	Median Age		*p*-Value
		≥ median age	< median age	
**MMP-7**	Tumor **Low**	11	14	0.40
	Tumor **High**	14	11	
	Stroma **Low**	12	13	0.77
	Stroma **High**	13	12	
	Serum **Low**	15	9	0.043
	Serum **High**	8	16	
**MMP-9**	Tumor **Low**	12	13	0.77
	Tumor **High**	13	12	
	Stroma **Low**	13	12	0.77
	Stroma **High**	12	13	
	Serum **Low**	11	14	0.57
	Serum **High**	12	11	
**MMP-10**	Tumor **Low**	14	11	0.40
	Tumor **High**	11	14	
	Stroma **Low**	14	13	0.77
	Stroma **High**	11	12	
	Serum **Low**	14	10	0.15
	Serum **High**	9	15	
**MMP-3**	Serum **Low**	13	11	0.39
	Serum **High**	10	14	
**MMP-12**	Serum **Low**	14	11	0.24
	Serum **High**	9	14	

Supplementary Table S6. Chi square (χ^2) test to evaluate an association between gender and angiogenic cytokines/MMPs derived from tumor cells, tumor-associated stromal cells or serum. *P*-values of all statistical tests were two-sided and $p \leq 0.001$ (after Bonferroni correction) were considered to be significant.

Cytokines/MMPs	Compartment	Gender		*p*-Value
		Male	Female	
**Angiopoietin-2**	Tumor **Low**	19	11	0.35
	Tumor **High**	10	10	
	Stroma **Low**	14	12	0.54
	Stroma **High**	15	9	
	Serum **Low**	13	11	0.77
	Serum **High**	14	10	
**Follistatin**	Tumor **Low**	14	11	0.77
	Tumor **High**	15	10	
	Stroma **Low**	12	13	0.15
	Stroma **High**	17	8	
	Serum **Low**	14	10	0.77
	Serum **High**	13	11	
**G-CSF**	Tumor **Low**	16	9	0.48
	Tumor **High**	13	11	
	Stroma **Low**	14	11	0.77
	Stroma **High**	15	10	
	Serum **Low**	13	11	0.77
	Serum **High**	14	10	
**HGF**	Tumor **Low**	17	8	0.15
	Tumor **High**	12	13	
	Stroma **Low**	15	10	0.77
	Stroma **High**	14	11	
	Serum **Low**	13	11	0.77
	Serum **High**	14	10	
**IL-8**	Tumor **Low**	15	10	0.77
	Tumor **High**	14	11	
	Stroma **Low**	14	11	0.77
	Stroma **High**	15	10	
	Serum **Low**	12	12	0.38
	Serum **High**	15	9	

(*Continued*)

Cytokines/MMPs	Compartment	Gender		*p*-Value
		Male	Female	
**Leptin**	Tumor **Low**	17	9	0.27
	Tumor **High**	12	12	
	Stroma **Low**	16	10	0.60
	Stroma **High**	13	11	
	Serum **Low**	21	4	**0.0001**
	Serum **High**	6	17	
**PDGF-BB**	Tumor **Low**	16	9	0.39
	Tumor **High**	13	12	
	Stroma **Low**	16	9	0.39
	Stroma **High**	13	12	
	Serum **Low**	13	11	0.77
	Serum **High**	14	10	
**PECAM-1**	Tumor **Low**	17	8	0.15
	Tumor **High**	12	13	
	Stroma **Low**	13	12	0.39
	Stroma **High**	16	9	
	Serum **Low**	13	11	0.77
	Serum **High**	14	10	
**VEGF**	Tumor **Low**	17	8	0.15
	Tumor **High**	12	13	
	Stroma **Low**	17	8	0.15
	Stroma **High**	12	13	
	Serum **Low**	13	11	0.77
	Serum **High**	14	10	
**MMP-1**	Tumor **Low**	15	10	0.77
	Tumor **High**	14	11	
	Stroma **Low**	15	10	0.77
	Stroma **High**	14	11	
	Serum **Low**	13	11	0.77
	Serum **High**	14	10	
**MMP-2**	Tumor **Low**	17	9	0.27
	Tumor **High**	12	12	
	Stroma **Low**	17	9	0.27
	Stroma **High**	12	12	
	Serum **Low**	10	14	0.042
	Serum **High**	17	7	

(*Continued*)

Cytokines/MMPs	Compartment	Gender		*p*-Value
		Male	Female	
**MMP-7**	Tumor **Low**	16	9	0.39
	Tumor **High**	13	12	
	Stroma **Low**	15	10	0.77
	Stroma **High**	14	11	
	Serum **Low**	13	11	0.77
	Serum **High**	14	10	
**MMP-9**	Tumor **Low**	16	9	0.32
	Tumor **High**	12	12	
	Stroma **Low**	16	9	0.39
	Stroma **High**	13	12	
	Serum **Low**	16	9	0.26
	Serum **High**	12	12	
**MMP-10**	Tumor **Low**	13	12	0.39
	Tumor **High**	16	9	
	Stroma **Low**	16	11	0.85
	Stroma **High**	13	10	
	Serum **Low**	12	12	0.38
	Serum **High**	15	9	
**MMP-3**	Serum **Low**	13	11	0.77
	Serum **High**	14	10	
**MMP-12**	Serum **Low**	16	9	0.26
	Serum **High**	11	12	

Supplementary Table S7. Chi square (χ^2) test to evaluate an association between tumor stage (pT) and angiogenic cytokines/MMPs derived from tumor cells, tumor-associated stromal cells or serum. *P*-values of all statistical tests were two-sided and $\rho \leq 0.001$ (after Bonferroni correction) were considered to be significant.

Cytokines/MMPs	Compartment	Local tumor stage		*p*-Value
		pT3	pT4	
**Angiopoietin-2**	Tumor **Low**	29	1	0.4
	Tumor **High**	20	0	
	Stroma **Low**	25	1	0.33
	Stroma **High**	24	0	
	Serum **Low**	23	1	0.31
	Serum **High**	24	0	
**Follistatin**	Tumor **Low**	24	1	0.31
	Tumor **High**	25	0	
	Stroma **Low**	24	1	0.31
	Stroma **High**	25	0	
	Serum **Low**	23	1	0.31
	Serum **High**	24	0	
**G-CSF**	Tumor **Low**	24	1	0.32
	Tumor **High**	24	0	
	Stroma **Low**	24	1	0.31
	Stroma **High**	25	0	
	Serum **Low**	23	1	0.31
	Serum **High**	24	0	
**HGF**	Tumor **Low**	24	1	0.31
	Tumor **High**	25	0	
	Stroma **Low**	24	1	0.31
	Stroma **High**	25	0	
	Serum **Low**	24	0	0.31
	Serum **High**	23	1	
**IL-8**	Tumor **Low**	24	1	0.31
	Tumor **High**	25	0	
	Stroma **Low**	24	1	0.32
	Stroma **High**	24	0	
	Serum **Low**	24	0	0.31
	Serum **High**	23	1	

(*Continued*)

Cytokines/MMPs	Compartment	Local tumor stage		*p*-Value
		pT3	pT4	
**Leptin**	Tumor **Low**	26	0	0.29
	Tumor **High**	23	1	
	Stroma **Low**	25	1	0.33
	Stroma **High**	24	0	
	Serum **Low**	24	1	0.33
	Serum **High**	23	0	
**PDGF-BB**	Tumor **Low**	24	1	0.31
	Tumor **High**	25	0	
	Stroma **Low**	24	1	0.31
	Stroma **High**	25	0	
	Serum **Low**	23	1	0.31
	Serum **High**	24	0	
**PECAM-1**	Tumor **Low**	24	1	0.31
	Tumor **High**	25	0	
	Stroma **Low**	24	1	0.31
	Stroma **High**	25	0	
	Serum **Low**	23	1	0.31
	Serum **High**	24	0	
**VEGF**	Tumor **Low**	24	1	0.31
	Tumor **High**	25	0	
	Stroma **Low**	24	1	0.31
	Stroma **High**	25	0	
	Serum **Low**	24	0	0.31
	Serum **High**	23	1	
**MMP-1**	Tumor **Low**	24	1	0.31
	Tumor **High**	25	0	
	Stroma **Low**	24	1	0.31
	Stroma **High**	25	0	
	Serum **Low**	24	0	0.31
	Serum **High**	23	1	
**MMP-2**	Tumor **Low**	25	1	0.33
	Tumor **High**	24	0	
	Stroma **Low**	24	1	0.31
	Stroma **High**	25	0	
	Serum **Low**	24	0	0.31
	Serum **High**	23	1	

(*Continued*)

Cytokines/MMPs	Compartment	Local tumor stage		*p*-Value
		pT3	pT4	
**MMP-7**	Tumor **Low**	24	1	0.31
	Tumor **High**	25	0	
	Stroma **Low**	25	1	0.31
	Stroma **High**	24	0	
	Serum **Low**	24	0	0.31
	Serum **High**	23	1	
**MMP-9**	Tumor **Low**	24	1	0.32
	Tumor **High**	24	0	
	Stroma **Low**	25	0	0.31
	Stroma **High**	24	1	
	Serum **Low**	24	1	0.33
	Serum **High**	23	0	
**MMP-10**	Tumor **Low**	24	1	0.31
	Tumor **High**	25	0	
	Stroma **Low**	26	1	0.35
	Stroma **High**	23	0	
	Serum **Low**	24	0	0.31
	Serum **High**	23	1	
**MMP-3**	Serum **Low**	24	0	0.31
	Serum **High**	23	1	
**MMP-12**	Serum **Low**	25	0	0.29
	Serum **High**	22	1	

Supplementary Table S8. Chi square (χ^2) test to evaluate an association between lymph node status (pN) and angiogenic cytokines/MMPs derived from tumor cells, tumor-associated stromal cells or serum. *P*-values of all statistical tests were two-sided and $\rho \leq 0.001$ (after Bonferroni correction) were considered to be significant.

Cytokines/MMPs	Compartment	Lymph node status		*P*-Value
		pN0	pN1	
**Angiopoietin-2**	Tumor **Low**	5	25	0.76
	Tumor **High**	4	16	
	Stroma **Low**	5	21	0.81
	Stroma **High**	4	20	
	Serum **Low**	5	19	0.71
	Serum **High**	4	20	
**Follistatin**	Tumor **Low**	7	18	0.66
	Tumor **High**	2	23	
	Stroma **Low**	6	19	0.27
	Stroma **High**	3	22	
	Serum **Low**	3	21	0.27
	Serum **High**	6	18	
**G-CSF**	Tumor **Low**	3	22	0.24
	Tumor **High**	6	18	
	Stroma **Low**	4	21	0.71
	Stroma **High**	5	20	
	Serum **Low**	3	21	0.27
	Serum **High**	6	18	
**HGF**	Tumor **Low**	4	21	0.71
	Tumor **High**	5	20	
	Stroma **Low**	5	20	0.71
	Stroma **High**	4	21	
	Serum **Low**	3	21	0.27
	Serum **High**	6	18	
**IL-8**	Tumor **Low**	4	21	0.71
	Tumor **High**	5	20	
	Stroma **Low**	6	19	0.27
	Stroma **High**	3	22	
	Serum **Low**	3	21	0.27
	Serum **High**	6	18	

(*Continued*)

Cytokines/MMPs	Compartment	Lymph node status		*P*-Value
		pN0	pN1	
**Leptin**	Tumor **Low**	6	20	0.33
	Tumor **High**	3	21	
	Stroma **Low**	6	20	0.33
	Stroma **High**	3	21	
	Serum **Low**	3	22	0.21
	Serum **High**	6	17	
**PDGF-BB**	Tumor **Low**	5	20	0.71
	Tumor **High**	4	21	
	Stroma **Low**	6	19	0.27
	Stroma **High**	3	22	
	Serum **Low**	4	20	0.71
	Serum **High**	5	19	
**PECAM-1**	Tumor **Low**	3	22	0.21
	Tumor **High**	6	19	
	Stroma **Low**	5	20	0.71
	Stroma **High**	4	21	
	Serum **Low**	4	20	0.71
	Serum **High**	5	19	
**VEGF**	Tumor **Low**	6	19	0.27
	Tumor **High**	3	22	
	Stroma **Low**	6	19	0.27
	Stroma **High**	3	22	
	Serum **Low**	3	21	0.27
	Serum **High**	6	18	
**MMP-1**	Tumor **Low**	4	21	0.71
	Tumor **High**	5	20	
	Stroma **Low**	4	21	0.71
	Stroma **High**	5	20	
	Serum **Low**	5	19	0.71
	Serum **High**	4	20	
**MMP-2**	Tumor **Low**	2	24	0.048
	Tumor **High**	7	17	
	Stroma **Low**	3	22	0.27
	Stroma **High**	6	19	
	Serum **Low**	6	18	0.27
	Serum **High**	3	21	

(*Continued*)

Cytokines/MMPs	Compartment	Lymph node status		*P*-Value
		pN0	pN1	
**MMP-7**	Tumor **Low**	6	19	0.27
	Tumor **High**	3	22	
	Stroma **Low**	6	19	0.27
	Stroma **High**	3	22	
	Serum **Low**	4	20	0.71
	Serum **High**	5	19	
**MMP-9**	Tumor **Low**	4	21	0.66
	Tumor **High**	5	19	
	Stroma **Low**	5	20	0.71
	Stroma **High**	4	21	
	Serum **Low**	6	19	0.33
	Serum **High**	3	20	
**MMP-10**	Tumor **Low**	4	21	0.71
	Tumor **High**	5	20	
	Stroma **Low**	6	21	0.40
	Stroma **High**	3	20	
	Serum **Low**	6	18	0.27
	Serum **High**	3	21	
**MMP-3**	Serum **Low**	4	20	0.71
	Serum **High**	5	19	
**MMP-12**	Serum **Low**	5	20	0.82
	Serum **High**	4	19	

Supplementary Table S9. Chi square (χ^2) test to evaluate an association between the prescence of synchronous distant metastases (M0) and angiogenic cytokines/MMPs derived from tumor cells, tumor-associated stromal cells or serum. *P*-values of all statistical tests were two-sided and $\rho \leq 0.001$ (after Bonferroni correction) were considered to be significant.

Cytokines/MMPs	Compartment	Distant metastases		*p*-Value
		M0	M1	
**Angiopoietin-2**	Tumor **Low**	27	3	0.52
	Tumor **High**	19	1	
	Stroma **Low**	23	3	0.34
	Stroma **High**	23	1	
	Serum **Low**	23	1	0.55
	Serum **High**	22	2	
**Follistatin**	Tumor **Low**	22	3	0.29
	Tumor **High**	24	1	
	Stroma **Low**	22	3	0.29
	Stroma **High**	24	1	
	Serum **Low**	22	2	0.55
	Serum **High**	23	1	
**G-CSF**	Tumor **Low**	23	2	0.96
	Tumor **High**	22	2	
	Stroma **Low**	23	2	1.00
	Stroma **High**	23	2	
	Serum **Low**	23	1	0.55
	Serum **High**	22	2	
**HGF**	Tumor **Low**	24	1	0.29
	Tumor **High**	22	3	
	Stroma **Low**	23	2	1.00
	Stroma **High**	23	2	
	Serum **Low**	23	1	0.55
	Serum **High**	22	2	
**IL-8**	Tumor **Low**	24	1	0.29
	Tumor **High**	22	3	
	Stroma **Low**	24	1	0.29
	Stroma **High**	22	3	
	Serum **Low**	23	1	0.55
	Serum **High**	22	2	

(*Continued*)

Cytokines/MMPs	Compartment	Distant metastases		*p*-Value
		M0	M1	
**Leptin**	Tumor **Low**	25	1	0.26
	Tumor **High**	21	3	
	Stroma **Low**	24	2	0.93
	Stroma **High**	22	2	
	Serum **Low**	22	3	0.08
	Serum **High**	23	0	
**PDGF-BB**	Tumor **Low**	23	2	1.00
	Tumor **High**	23	2	
	Stroma **Low**	23	2	1.00
	Stroma **High**	23	2	
	Serum **Low**	24	0	0.074
	Serum **High**	21	3	
**PECAM-1**	Tumor **Low**	22	3	0.30
	Tumor **High**	24	1	
	Stroma **Low**	23	2	1.00
	Stroma **High**	23	2	
	Serum **Low**	22	2	0.55
	Serum **High**	23	1	
**VEGF**	Tumor **Low**	23	2	1.00
	Tumor **High**	23	2	
	Stroma **Low**	23	2	1.00
	Stroma **High**	23	2	
	Serum **Low**	24	0	0.074
	Serum **High**	21	3	
**MMP-1**	Tumor **Low**	22	3	0.30
	Tumor **High**	24	1	
	Stroma **Low**	22	3	0.30
	Stroma **High**	24	1	
	Serum **Low**	22	2	0.55
	Serum **High**	23	1	
**MMP-2**	Tumor **Low**	25	1	0.26
	Tumor **High**	21	3	
	Stroma **Low**	23	2	1.00
	Stroma **High**	23	2	
	Serum **Low**	23	1	0.55
	Serum **High**	22	2	

(*Continued*)

Cytokines/MMPs	Compartment	Distant metastases		*p*-Value
		M0	M1	
**MMP-7**	Tumor **Low**	23	2	1.00
	Tumor **High**	23	2	
	Stroma **Low**	22	3	0.30
	Stroma **High**	24	1	
	Serum **Low**	23	1	0.55
	Serum **High**	22	2	
**MMP-9**	Tumor **Low**	23	2	0.96
	Tumor **High**	22	2	
	Stroma **Low**	24	1	0.30
	Stroma **High**	22	3	
	Serum **Low**	23	2	0.60
	Serum **High**	22	1	
**MMP-10**	Tumor **Low**	24	1	0.30
	Tumor **High**	22	3	
	Stroma **Low**	26	1	0.22
	Stroma **High**	20	3	
	Serum **Low**	22	2	0.55
	Serum **High**	23	1	
**MMP-3**	Serum **Low**	24	0	0.074
	Serum **High**	21	3	
**MMP-12**	Serum **Low**	24	1	0.50
	Serum **High**	21	2	

Supplementary Table S10. Chi square (χ^2) test to evaluate an association between the grade of tumor differentiation (G) and angiogenic cytokines/MMPs derived from tumor cells, tumor-associated stromal cells or serum. *P*-values of all statistical tests were two-sided and $\rho \leq 0.001$ (after Bonferroni correction) were considered to be significant.

Cytokines/MMPs	Compartment	Tumor grade		*p*-Value
		G2	G3	
**Angiopoietin-2**	Tumor **Low**	17	13	0.64
	Tumor **High**	10	10	
	Stroma **Low**	12	14	0.25
	Stroma **High**	15	9	
	Serum **Low**	15	9	0.25
	Serum **High**	11	13	
**Follistatin**	Tumor **Low**	14	11	0.77
	Tumor **High**	13	12	
	Stroma **Low**	12	13	0.40
	Stroma **High**	15	10	
	Serum **Low**	13	11	1.00
	Serum **High**	13	11	
**G-CSF**	Tumor **Low**	13	12	0.88
	Tumor **High**	13	11	
	Stroma **Low**	13	12	0.77
	Stroma **High**	14	11	
	Serum **Low**	11	13	0.25
	Serum **High**	15	9	
**HGF**	Tumor **Low**	13	12	0.77
	Tumor **High**	14	11	
	Stroma **Low**	13	12	0.77
	Stroma **High**	14	11	
	Serum **Low**	12	12	0.56
	Serum **High**	14	10	
**IL-8**	Tumor **Low**	15	10	0.40
	Tumor **High**	12	13	
	Stroma **Low**	16	9	0.16
	Stroma **High**	11	14	
	Serum **Low**	13	11	1.00
	Serum **High**	13	11	

(*Continued*)

Cytokines/MMPs	Compartment	Tumor grade		p-Value
		G2	G3	
**Leptin**	Tumor **Low**	17	9	0.09
	Tumor **High**	10	14	
	Stroma **Low**	12	14	0.25
	Stroma **High**	15	9	
	Serum **Low**	11	14	0.14
	Serum **High**	15	8	
**PDGF-BB**	Tumor **Low**	12	13	0.40
	Tumor **High**	15	10	
	Stroma **Low**	14	11	0.77
	Stroma **High**	13	12	
	Serum **Low**	12	12	0.56
	Serum **High**	14	10	
**PECAM-1**	Tumor **Low**	13	12	0.77
	Tumor **High**	14	11	
	Stroma **Low**	14	11	0.77
	Stroma **High**	13	12	
	Serum **Low**	9	15	0.02
	Serum **High**	17	7	
**VEGF**	Tumor **Low**	12	13	0.40
	Tumor **High**	15	10	
	Stroma **Low**	12	13	0.40
	Stroma **High**	15	10	
	Serum **Low**	14	10	0.56
	Serum **High**	12	12	
**MMP-1**	Tumor **Low**	11	14	0.16
	Tumor **High**	16	9	
	Stroma **Low**	10	15	0.047
	Stroma **High**	17	8	
	Serum **Low**	13	11	1.00
	Serum **High**	13	11	
**MMP-2**	Tumor **Low**	13	13	0.56
	Tumor **High**	14	10	
	Stroma **Low**	12	13	0.40
	Stroma **High**	15	10	
	Serum **Low**	16	8	0.08
	Serum **High**	10	14	

(*Continued*)

Cytokines/MMPs	Compartment	Tumor grade		*p*-Value
		G2	G3	
**MMP-7**	Tumor **Low**	15	10	0.40
	Tumor **High**	12	13	
	Stroma **Low**	12	13	0.40
	Stroma **High**	15	10	
	Serum **Low**	13	11	1.00
	Serum **High**	13	11	
**MMP-9**	Tumor **Low**	15	10	0.32
	Tumor **High**	11	13	
	Stroma **Low**	17	8	0.047
	Stroma **High**	10	15	
	Serum **Low**	12	13	0.37
	Serum **High**	14	9	
**MMP-10**	Tumor **Low**	16	9	0.16
	Tumor **High**	11	14	
	Stroma **Low**	14	13	0.74
	Stroma **High**	13	10	
	Serum **Low**	16	8	0.08
	Serum **High**	10	14	
**MMP-3**	Serum **Low**	16	8	0.08
	Serum **High**	10	14	
**MMP-12**	Serum **Low**	13	12	0.75
	Serum **High**	13	10	

Supplementary Table S11. Chi square (χ^2) test to evaluate an association between the resection status (R) and angiogenic cytokines/MMPs derived from tumor cells, tumor-associated stromal cells or serum. *P*-values of all statistical tests were two-sided and $\rho \leq 0.001$ (after Bonferroni correction) were considered to be significant.

**Cytokines/MMPs**	**Compartment**	**Resection status**		***p*-Value**
**Angiopoietin-2**	Tumor **Low**	4	26	0.87
	Tumor **High**	3	17	
	Stroma **Low**	4	22	0.77
	Stroma **High**	3	21	
	Serum **Low**	3	21	0.68
	Serum **High**	4	20	
**Follistatin**	Tumor **Low**	5	20	0.22
	Tumor **High**	2	23	
	Stroma **Low**	4	21	0.68
	Stroma **High**	3	22	
	Serum **Low**	3	21	0.68
	Serum **High**	4	20	
**G-CSF**	Tumor **Low**	1	24	0.036
	Tumor **High**	6	18	
	Stroma **Low**	3	21	0.68
	Stroma **High**	4	20	
	Serum **Low**	1	23	0.04
	Serum **High**	6	18	
**HGF**	Tumor **Low**	3	22	0.68
	Tumor **High**	4	21	
	Stroma **Low**	5	20	0.22
	Stroma **High**	2	23	
	Serum **Low**	3	21	0.68
	Serum **High**	4	20	
**IL-8**	Tumor **Low**	3	22	0.68
	Tumor **High**	4	21	
	Stroma **Low**	4	21	0.68
	Stroma **High**	3	20	
	Serum **Low**	4	20	0.68
	Serum **High**	3	21	

(*Continued*)

Cytokines/MMPs	Compartment	Resection status		p-Value
**Leptin**	Tumor **Low**	7	19	0.006
	Tumor **High**	0	24	
	Stroma **Low**	5	21	0.27
	Stroma **High**	2	22	
	Serum **Low**	3	22	0.59
	Serum **High**	4	19	
**PDGF-BB**	Tumor **Low**	3	22	0.68
	Tumor **High**	4	21	
	Stroma **Low**	4	21	0.68
	Stroma **High**	3	22	
	Serum **Low**	3	21	0.68
	Serum **High**	4	20	
**PECAM-1**	Tumor **Low**	1	24	0.04
	Tumor **High**	6	19	
	Stroma **Low**	2	23	0.22
	Stroma **High**	5	20	
	Serum **Low**	2	22	0.22
	Serum **High**	5	19	
**VEGF**	Tumor **Low**	5	20	0.22
	Tumor **High**	2	23	
	Stroma **Low**	5	20	0.22
	Stroma **High**	2	23	
	Serum **Low**	2	22	0.22
	Serum **High**	5	19	
**MMP-1**	Tumor **Low**	2	23	0.22
	Tumor **High**	5	20	
	Stroma **Low**	3	22	0.68
	Stroma **High**	4	21	
	Serum **Low**	4	20	0.68
	Serum **High**	3	21	
**MMP-2**	Tumor **Low**	3	23	0.60
	Tumor **High**	4	20	
	Stroma **Low**	3	22	0.68
	Stroma **High**	4	21	
	Serum **Low**	3	20	0.68
	Serum **High**	4	21	

(*Continued*)

Cytokines/MMPs	Compartment	Resection status		*p*-Value
**MMP-7**	Tumor **Low**	4	21	0.68
	Tumor **High**	3	22	
	Stroma **Low**	4	21	0.68
	Stroma **High**	3	22	
	Serum **Low**	4	20	0.68
	Serum **High**	3	21	
**MMP-9**	Tumor **Low**	2	23	0.20
	Tumor **High**	5	19	
	Stroma **Low**	3	22	0.68
	Stroma **High**	4	21	
	Serum **Low**	3	22	0.60
	Serum **High**	4	19	
**MMP-10**	Tumor **Low**	3	22	0.68
	Tumor **High**	4	21	
	Stroma **Low**	3	22	0.68
	Stroma **High**	4	21	
	Serum **Low**	5	22	0.32
	Serum **High**	2	21	
**MMP-3**	Serum **Low**	3	21	0.68
	Serum **High**	4	20	
**MMP-12**	Serum **Low**	5	20	0.26
	Serum **High**	2	21	
